# Laser Capture Microdissection of Single Neurons with Morphological Visualization Using Fluorescent Proteins Fused to Transmembrane Proteins

**DOI:** 10.1523/ENEURO.0275-20.2021

**Published:** 2021-09-02

**Authors:** Ching Ching Chang, Hai Tarng Chong, Ayumu Tashiro (田代 歩)

**Affiliations:** School of Biological Sciences, Nanyang Technological University, Singapore 308232

**Keywords:** channelrhodopsin, gene expression, halorhodopsin, optogenetics, viral vector

## Abstract

Gene expression analysis in individual neuronal types helps in understanding brain function. Genetic methods expressing fluorescent proteins are widely used to label specific neuronal populations. However, because cell type specificity of genetic labeling is often limited, it is advantageous to combine genetic labeling with additional methods to select specific cell/neuronal types. Laser capture microdissection is one of such techniques with which one can select a specific cell/neuronal population based on morphological observation. However, a major issue is the disappearance of fluorescence signals during the tissue processing that is required for high-quality sample preparation. Here, we developed a simple, novel method in which fluorescence signals are preserved. We use genetic labeling with fluorescence proteins fused to transmembrane proteins, which shows highly stable fluorescence retention and allows for the selection of fluorescent neurons/cells based on morphology. Using this method in mice, we laser-captured neuronal somata and successfully isolated RNA. We determined that ∼100 cells are sufficient to obtain a sample required for downstream applications such as quantitative PCR. Capability to specifically microdissect targeted neurons was demonstrated by an ∼10-fold increase in mRNA for fluorescent proteins in visually identified neurons expressing the fluorescent proteins compared with neighboring cells not expressing it. We applied this method to validate virus-mediated single-cell knockout, which showed up to 92% reduction in knocked-out gene RNA compared with wild-type neurons. This method using fluorescent proteins fused to transmembrane proteins provides a new, simple solution to perform gene expression analysis in sparsely labeled neuronal/cellular populations, which is especially advantageous when genetic labeling has limited specificity.

## Significance Statement

Genetic labeling of specific cell types with reporter fluorescent proteins is widely used for gene expression analysis. However, because the specificity of genetic labeling is often limited, an additional method is required to collect samples from a specific cell type. Here, we developed a novel method to allow for the selection of a cell/neuronal type of interest based on morphology before performing sample collection using laser capture microdissection. Stable fluorescent signals from fluorescent proteins fused to transmembrane proteins allow us to morphologically select and laser capture hundreds of single neurons. This method would be applicable to genomic and transcriptomic analysis of different cell/neuronal types and could facilitate our understanding of brain functions.

## Introduction

Gene expression analysis of specific neuronal types has been successfully performed using genetic labeling with reporter fluorescent proteins and fluorescence-activated cell sorting (FACS; Xia et al., 2017; Daigle et al., 2018). However, one technical limitation is that genetic labeling often marks multiple neuronal and/or other cell types (van Praag et al., 2002; Balthasar et al., 2004; Lagace et al., 2007; Harris et al., 2014), whereas FACS cannot separate such unwanted, labeled cell types. In addition, enzyme-mediated cell dissociation procedures could affect viability and gene expression (Ho et al., 2018; Hussain et al., 2018; Reichard and Asosingh, 2019). An alternative method that allows for separating cell/neuronal types without tissue dissociation steps would be useful as a complementary method.

Laser capture microdissection is a technique that dissects a small piece of tissue or a single cell by a laser and collects them with gravity or the assistance of a laser. Instruments for laser capture microdissection are often equipped with a fluorescence light source (Emmert-Buck et al., 1996; Bonner et al., 1997; Fend and Raffeld, 2000). Therefore, one can observe the morphology of genetically labeled fluorescent cells and select a cell type of interest to capture. However, in practice, this is not a simple task. Commonly for laser capture microdissection, sections are prepared from freshly frozen tissue, rather than fixed tissue, to acquire high-quality RNA. In such sections, fluorescent proteins diffuse out from cells and their fluorescence signals disappear during tissue-processing procedures (Singh et al., 2019). Although an optimized protocol can improve the issue to some extent, the instability of fluorescence signals makes it difficult to use fluorescence as a guide to distinguish cell types for laser capture microdissection. A more stable fluorescence-labeling method would be valuable for solving this technical limitation. In this line, studies (Rossner et al., 2006; Chatzi et al., 2019) demonstrated that nuclear targeted fluorescent proteins preserves their signals. However, because only nuclei, but not processes/neurites, are visualized, their application to morphological identification of cell types is limited.

In the course of an experiment for some other purpose, we happened to find that the fluorescence signals from transmembrane proteins fused with fluorescent proteins were extremely stable and kept visualizing dendritic morphology in air-dried, nonfixed brain sections. We later found that similar preservation of fluorescence signals from transmembrane proteins fused with fluorescent proteins has been previously described in the context of flow cytometry of primary human fibroblasts (Kalejta et al., 1997). This observation made us reason that genetic labeling with fluorescent proteins fused to transmembrane proteins allows us to identify a cell type of interest based on their morphology and selectively capture them using laser capture microdissection. In this study, we tested this possibility and validated that the use of labeling with fluorescent proteins fused to transmembrane proteins allows us to select neurons over non-neuronal cells and perform downstream gene expression analyses.

## Materials and Methods

**Figure 1.**
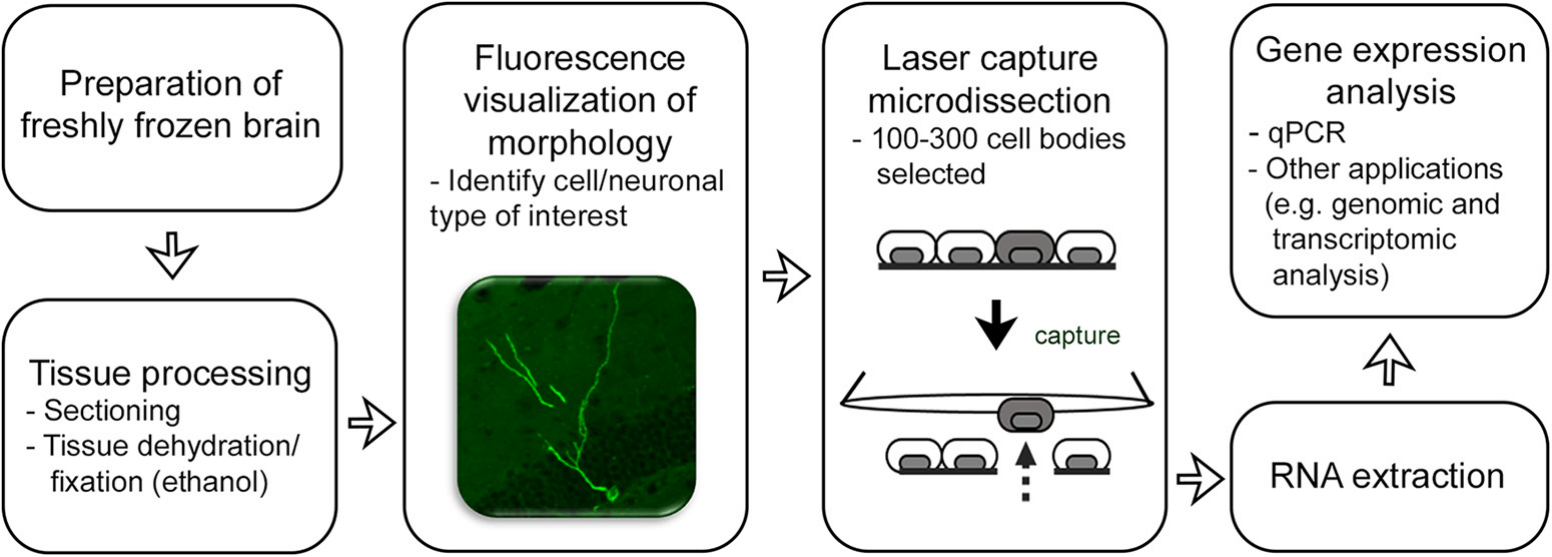
Flowchart of methodology.

A flowchart of the methodology is shown in Figure 1. We freshly froze brains after retrieving them from the skull. We sectioned the brains, mounted sections on the glass slides and dehydrated/fixed them with ethanol. We distinguished neurons from glial cells based on their dendritic morphology under a fluorescence microscope, and then harvested the cell bodies of the selected neurons with laser capture microdissection. A total of 100–300 microdissected neurons were pooled for RNA extraction. As examples of downstream gene expression analyses, we performed quantitative PCR (qPCR) analysis after cDNA amplification.

### Mice

We used 6- to 11-week-old C57BL/6 or transgenic mice of either sex, housed in a 12 h dark/light cycle with *ad libitum* access to water and food. Transgenic lines used were floxed NR1 mice (B6.129S4-Grin1^tm2Stl/J^; Tsien et al., 1996) and Pomc-ChR2 mice, which were produced by crossing two transgenic lines: STOCK Tg(Pomc1-cre)16Lowl/J (back-crossed with C57BL/6 mice; Balthasar et al., 2004) and B6.Cg-Gt(ROSA)26Sortm32(CAG-COP4*H134R/EYFP)Hze/J (Madisen et al., 2012). All three transgenic lines were originally purchased from The Jackson Laboratory and bred in our local facilities. All animal procedures were performed according to the approval from the Institutional Animal Care and Use Committee at Nanyang Technological University.

### Preparation of brain sections and tissue processing

Mice were deeply anesthetized with isoflurane. Mice were decapitated, and their brains were dissected out from the skull within 2 min. The brains were transferred onto an embedding mold and frozen in embedding medium (O.C.T. Compound, Sakura Finetek) with isopentane on dry ice, which was completed within an additional 1–3 min. Brains were stored at −80°C until cryosectioning. Immediately before cryosectioning, brains were kept at −20°C for at least 30 min. The 10- to 12-μm-thick sections were produced using a cryostat microtome at −20°C. Brain sections were mounted on glass slides and air dried for 5 min. Sections were dehydrated/fixed in increasing concentrations of ethanol (75%, 95%, and then 100%) for 30 s each, followed by xylene for 1 min. We performed these procedures immediately before fluorescence visualization and laser capture microdissection.

### Fluorescence visualization and laser capture microdissection

We used the PALM MicroBeam System (Zeiss) equipped with a fluorescence microscope. Each fluorescently labeled cell was selected under the microscope with an LD Plan-Neofluar objective lens (40×/0.6 numerical aperture), and laser captured into an AdhesiveCap 500 opaque (Zeiss). A sample of up to ∼100 cells were collected in a single AdhesiveCap and lysed with cell lysis buffer. When the collection took more than 1 h, we lysed cells to minimize sample degradation and continued collection in a new AdhesiveCap.

### RNA extraction

After cell lysis in the AdhesiveCaps, we extracted RNA using an Ambion RNA Isolation Kit (Thermo Fisher Scientific) according to the manufacturer protocol. When more than one Adhesivecap was used for sample collection, cell lysates from multiple AdhesiveCaps were pooled and passed through a single column. RNA concentration was measured using Quant-iT RiboGreen (Thermo Fisher Scientific).

### Quantitative PCR

Reverse transcription and cDNA amplification were performed using cDNA SMARTer PCR cDNA Synthesis Kit (Takara Bio) or GeneChip WT Pico Kit (Affymetrix). qPCR was performed with iTaq Universal SYBR Green Supermix (Bio-Rad) and StepOnePlus Real-Time PCR System (Thermo Fisher Scientific), using the following gene-specific primers: NR1 forward, TGTCCTGGGACTGACTACCC; NR1 reverse, CTGACCAGCAGGATGA; yellow fluorescent protein (YFP) forward, TATATCATGGCCGACAAGCA; YFP reverse, TGTTCTGCTGGTAGTGGTCG; doublecortin (DCX) forward, GAGTGCGCTACATTTATACCATTG; DCX reverse, TGACATTCTTGGTGTACTCAACCT; CaMKIIα forward, AGGATGAAGACACCAAAGTGC; CaMKIIα reverse, GGTTCAAAGGCTGTCATTCC; β-actin forward, AGCCATGTACGTAGCCATCC; and β-actin reverse, CTCTCAGCTGTGGTGGTGAA. Each sample was amplified in triplicate. We used mouse liver total DNA (see Fig. 4*D*) that was included in cDNA SMARTer PCR cDNA Synthesis Kit (Takara Bio).

### RNA integrity assay

The quality of RNA samples that we isolated was evaluated by a service provided by NovogeneAIT Genomics Singapore Pte Ltd, using an Agilent RNA 6000 Pico Kit and an Agilent 2100 Bioanalyzer. The quality of RNA was determined as the RNA integrity number (Schroeder et al., 2006).

### Preparation of viral vectors

We prepared the viral vectors as previously described (Tashiro et al., 2015a). We used Moloney murine leukemia virus-based retroviral vectors driving the expression of eNpHR3.0-YFP, mCherry, GFP-F, ChR2(SFFO), or bicistronically eNPHR3.0YFP and Cre under the control of the CAG promoter (Zhao et al., 2006) and an adeno-associated viral vector expressing mCherry-TRPV2 (transient receptor potential cation channel V2) under the control of the CaMKIIα promoter (Kitanishi et al., 2015).

### Stereotaxic injections

Mice were bilaterally injected with retroviral vectors (1.5 μl, titer: 1 × 10^7^ to 1 × 10^8^ colony-forming units/ml) into the dentate gyrus (coordinates: anteroposterior, 2 mm from the bregma; mediolateral, 1.5 mm from the bregma; dorsoventral, 2.3 mm from skull surface). Preparation and injection procedures were described previously (Tashiro et al., 2015a). For coinjection, a mixture of virus solutions was prepared with a 1:1 ratio in volume.

### Perfusion and fixation

**Figure 2.**
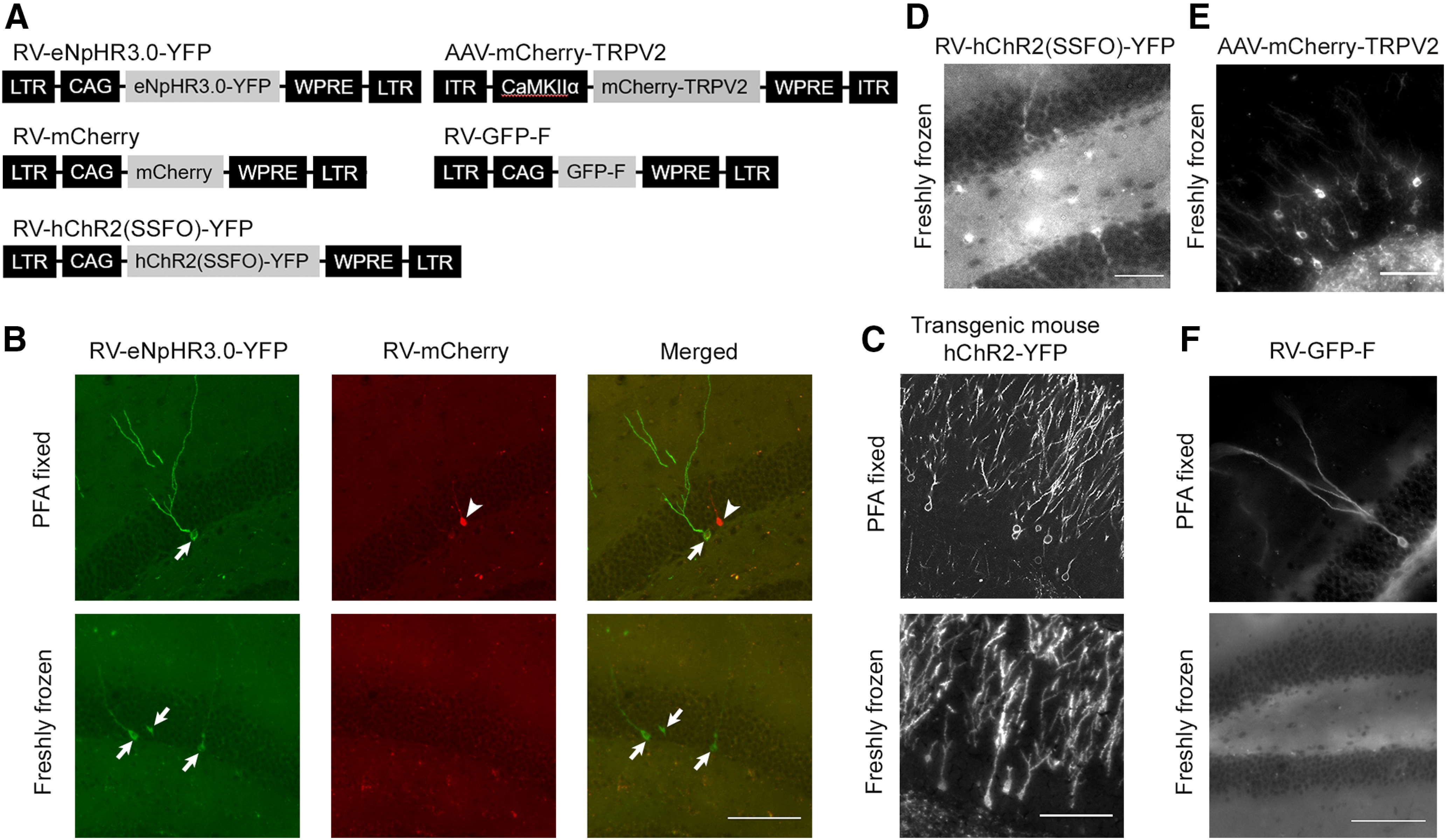
The signals of fluorescent proteins fused to transmembrane proteins are preserved after the standard tissue-processing procedure for laser capture microdissection. ***A***, Schematic structures of the viral vector genomes. ***B–F***, Images of fluorescently labeled granule cells in the dentate gyrus of mice injected with a mixture of two retroviral vectors expressing YFP-fused enhanced halorhodopsin, eNpHR3.0-YFP, or mCherry (***B***); Pomc-ChR2-YFP transgenic mice (***C***) and mice injected with a retroviral vector expressing ChR2(SSFO)-YFP (***D***); an adeno-associated viral vector expressing mCherry-TRPV2 (***E***) and a retroviral vector expressing a membrane-targeted form of GFP, GFP-F (***F***). Brain sections were prepared from paraformaldehyde fixed or freshly frozen brains and went through the standard tissue-processing procedure. Note that fluorescence signals of eNpHR3.0-YFP (***B***), ChR2-YFP (***C***), ChR2(SSFO)-YFP (***D***), and mCherry-TRPV2 (***E***), but not mCherry (***B***) and GFP-F (***F***), were preserved in sections prepared from freshly frozen brains. Arrows and arrowheads in ***B*** indicate somata labeled by eNpHR3.0-YFP and mCherry. RV, Retroviral vector; AAV, adeno-associated viral vector; LTR, long-terminal repeat; CAG, CAG promoter; WPRE, Woodchuck hepatitis virus post-transcriptional regulatory element; CaMKIIα, the CaMKIIα promoter; ITR, inverted terminal repeat. Scale bars, 100 μm.

For the purpose of visualizing fluorescence expression in neurons (Fig. 2, see also Fig. 7*C*), brain sections were prepared from brains that were perfusion fixed with 4% paraformaldehyde. The procedure was previously described (Tashiro et al., 2015b). Brain sectioning was performed in the same way as described in the subsection Preparation of brain sections and tissue processing. Sections were mounted on glass slides with coverslips and antifade polyvinyl alcohol mounting medium with DABCO.

### Freeze–thaw cycle with HEK293 cell culture with or without cryoprotectant

We seeded 1 × 10^5^ HEK293 cells in wells in a 12-well plate. Next day, we transfected the cells with one of the plasmids listed below, with a calcium phosphate method with the following mixture: 500 ng of plasmid DNA in 640 μl of sterile Milli-Q water and 36 μl of 2 m CaCl_2_, with 100 μl of 2× HEBS (50 mM HEPES, 280 mM NaCl, 10 mM KCl, 1.5 mM Na_2_HPO_4_, 12 mM d-glucose, pH 7.05). The plasmid DNAs were retroviral transfer plasmids, RV-CAG-GFP, RV-CAG-mCherry, RV-CAG-eNpHR3.0-YFP, RV-CAG-ChR2-mCherry, and RV-CAG-ChR2(SSFO)-YFP.

Twenty-four hours after transfection, we removed culture media from the wells and washed twice with PBS. For cells with cryoprotectant, we applied 500 μl of 10% DMSO/90% fetal bovine serum on top of the cells before a freeze–thaw cycle. For cells without cryoprotectant, we air dried and froze/thawed cells without cryoprotectant. We froze the cells by keeping the 12-well plate in a −80°C freezer for 30 min. We then thawed them at room temperature and performed imaging immediately.

## Results

### Fluorescence signals from fluorescent proteins fused to transmembrane proteins are preserved after tissue processing

In preliminary experiments, we tried to laser capture single neurons labeled with regular fluorescent proteins, such as the green fluorescent protein (GFP), which is soluble in the cytoplasm. After tissue-processing procedures, fluorescence signals disappeared in sections prepared from freshly frozen brains, and our attempt failed. However, in the course of an experiment for some other purpose, we happened to find that fluorescence signals from fluorescent proteins fused to transmembrane proteins are extremely stable even in air-dried, nonfixed brain sections prepared from freshly frozen brains. Under the same conditions, the fluorescence signals of regular fluorescent proteins diffuse and dissipate quickly in the order of seconds to minutes. This preliminary finding led us to test whether labeling with fluorescent proteins fused to transmembrane proteins is preserved after the standard tissue-processing procedure for laser capture microdissection.

Figure 2 illustrates the instability of cytoplasmic fluorescent proteins and the stability of fluorescent proteins fused to transmembrane proteins in sections from freshly frozen brains, which we processed by the standard protocol of tissue processing used for laser capture microdissection. We injected a mixture of two retroviral vectors into the dentate gyrus of adult mice (Fig. 2*A*). One of them was for expressing YFP fused to transmembrane proteins (enhanced halorhodopsin, eNpHR3.0), while the other was for expressing mCherry, which is cytosolic fluorescent proteins. Some of the mice were perfusion fixed with 4% paraformaldehyde, while the brains of the other mice were freshly frozen without fixation. All brains were then subjected to the standard tissue-processing procedure. As expected, paraformaldehyde-fixed brain sections preserved both YFP and mCherry signals (Fig. 2*B*, top). On the other hand, in sections from freshly frozen brains, YFP signals were preserved while mCherry signals were completely lost (Fig. 2*B*, bottom). Thus, fluorescent proteins fused to transmembrane proteins, but not cytosolic fluorescent proteins, preserve the fluorescence signals after the standard tissue-processing procedure for laser capture microdissection. Importantly, the dendritic morphology of YFP^+^ neurons was preserved and clearly visualized in the sections from freshly frozen brains (Fig. 2*B*).

In addition to enhanced halorhodopsin, we tested fluorescent proteins fused to another type of transmembrane proteins, channelrhodopsin-2 (ChR2). We prepared brain sections from (1) Pomc-ChR2-YFP mice expressing ChR2 fused to YFP in a subset of granule cells in the dentate gyrus and (2) adult mice that were injected with retroviral vector expressing a stable step function opsin (SSFO) mutant of ChR2 fused to YFP (Fig. 2*A*) into the dentate gyrus. Then we performed tissue processing. As shown in Figure 2, *C* and *D*, fluorescence signals in neurons were preserved in freshly frozen sections.

ChR2 and eNpHR3.0 are structurally similar proteins. To test whether fusion to a different type of transmembrane proteins also preserves fluorescence signals, we prepared brain sections from mice injected with an adeno-associated viral vector expressing TRPV2 (Fig. 2*A*) into the dentate gyrus. As shown in Figure 2*E*, fluorescence signals were again preserved in freshly frozen sections.

To test whether membrane-targeted fluorescent proteins with a farnesylation signal are preserved after tissue processing, we injected a retroviral vector expressing GFP fused with a farnesylation signal (GFP-F; Moriyoshi et al., 1996; Jiang and Hunter, 1998; Fig. 2*A*) into the dentate gyrus of adult mice. In cells, a farnesyl group is added to GFP-F and facilitates the attachment to cellular membrane. Although sections from brains fixed with paraformaldehyde preserved clear fluorescence labeling of neurons (Fig. 2*F*, top), no fluorescence signal was detected in the sections from freshly frozen brains (Fig. 2*F*, bottom). Thus, membrane-targeted fluorescence proteins do not preserve its fluorescence signals through the standard tissue-processing procedure.

**Figure 3.**
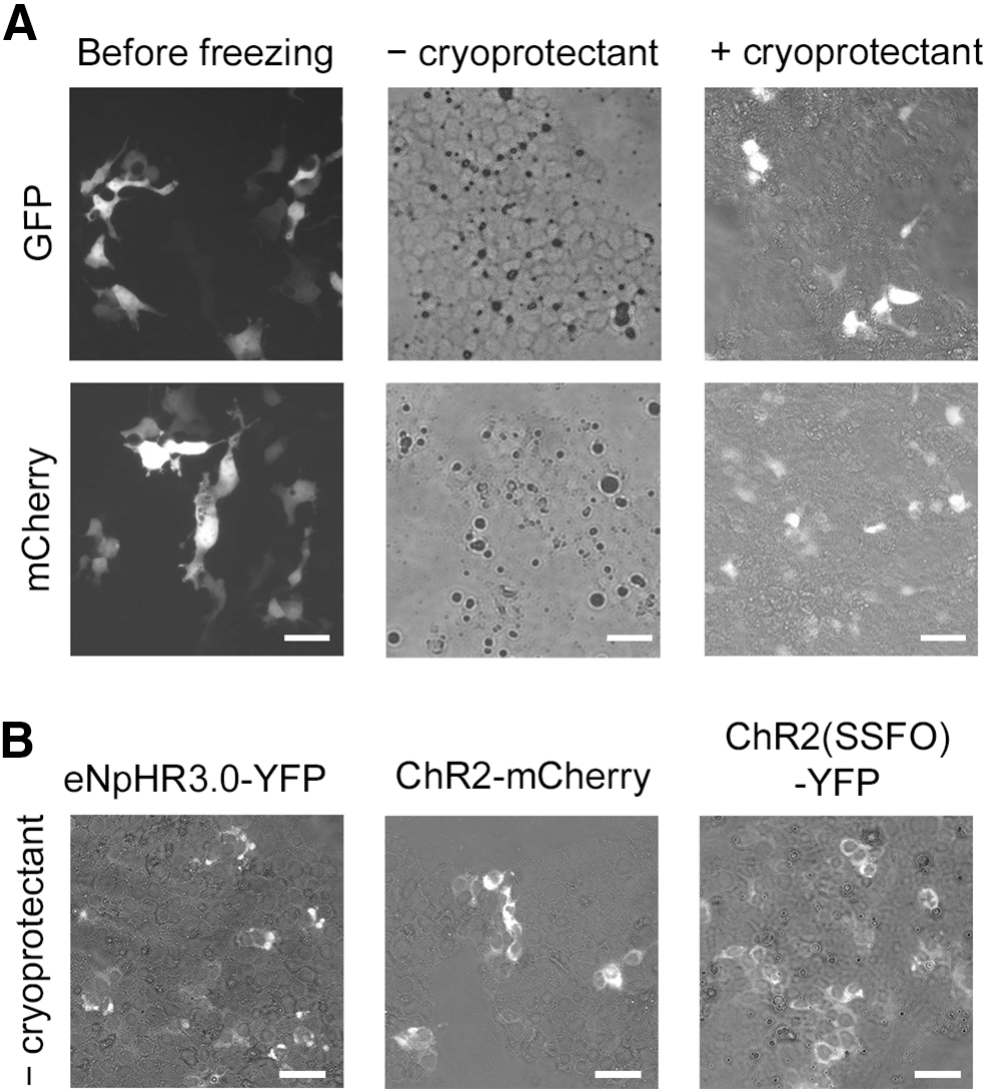
Cryoprotectant prevents the loss of fluorescence signals from regular fluorescent proteins in HEK293 cell culture after a freeze–thaw cycle. ***A***, Representative images showing fluorescence signals from regular, cytosolic fluorescent proteins (GFP and mCherry) after a freeze–thaw cycle with or without cryoprotectant. Fluorescence signals disappeared without cryoprotectant while cryoprotectant preserved fluorescence signals. ***B***, Representative images showing fluorescence signals from fluorescent proteins fused to transmembrane proteins [eNpHR3.0-YFP, ChR2-mCherry, ChR2(SSFO)-YFP]. Fluorescence signals were preserved without cryoprotectant after a freeze–thaw cycle. Scale bar, 50 μm.

We found that the loss of fluorescence signals from regular fluorescent proteins also occurs in HEK293 cells after freezing and thawing (Fig. 3*A*). However, when we added cryoprotectant (10% DMSO/90% fetal bovine serum), fluorescence signals were retained. This is presumably because cryoprotectant prevented the freeze–thaw cycle from rupturing the plasma membrane. In contrast, fluorescence signals from fluorescent proteins fused to transmembrane proteins were preserved after a freeze–thaw cycle without cryoprotectant (Fig. 3*B*). The results suggest that the breakage of plasma membrane caused by freezing causes the leakage of regular cytoplasmic fluorescent proteins but still keeps fluorescent proteins fused to transmembrane proteins in HEK293 cells, which is likely the same in brain sections.

### Laser capture microdissection of morphologically identified neurons

**Figure 4.**
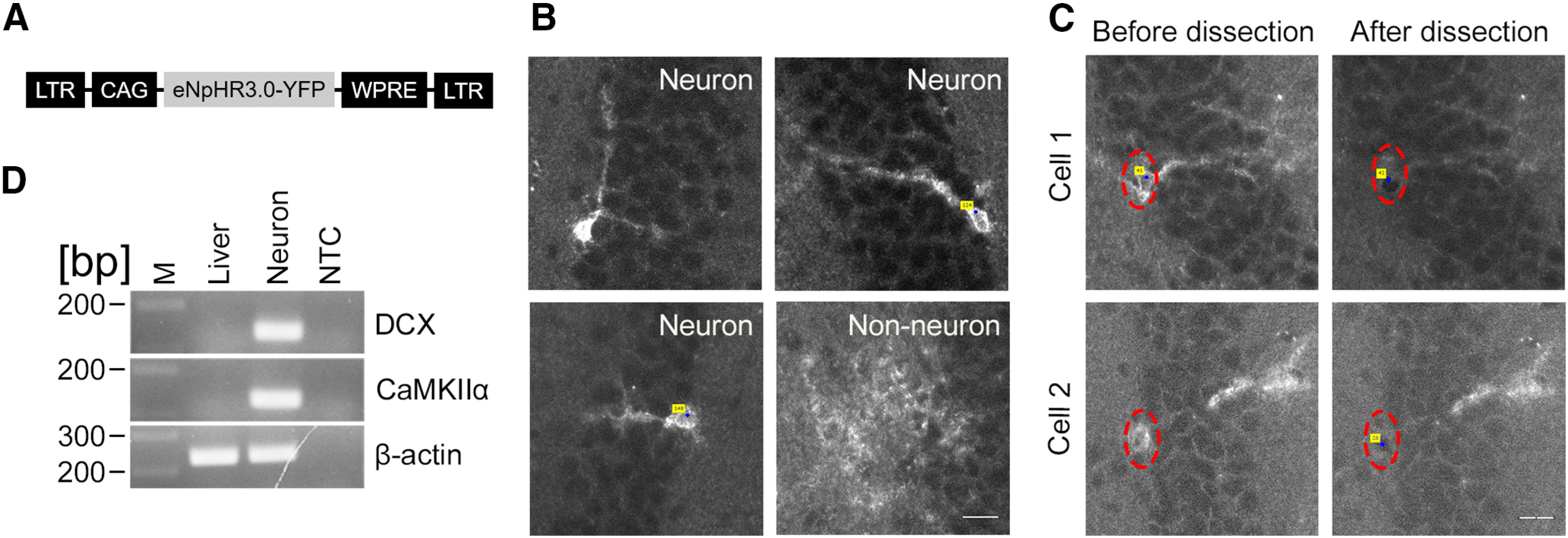
Laser capture microdissection of fluorescence labeled neurons. ***A***, A schematic structure of the viral vector genome. ***B***, Images of eNpHR3.0-YFP-expressing neurons and non-neuronal cells in the dentate gyrus of mice that were injected with a retroviral vector expressing eNpHR3.0-YFP. ***C***, Fluorescence images of sections before and after laser capture microdissection. Red dotted ovals show the location of somata of fluorescently labeled neurons selected for laser capture microdissection. Blue dots with yellow labels indicate the center of a selected area used for laser capture. Scale bar, 20 μm. ***D***, An electrophoresis image showing PCR amplification end products for the DCX, CaMKIIα, and β-actin genes. Note the high expression of neuronal markers in fluorescently labeled neurons compared with a total liver RNA sample where DCX and CaMKIIα were not detected. M, Molecular marker; NTC, nontemplate control.

Next, we performed laser capture microdissection of single neurons labeled with fluorescent proteins fused to transmembrane proteins. We injected a retroviral vector expressing eNpHR3.0-YFP into adult mice (Fig. 4*A*) and, after 14 d, harvested their brains and prepared brain sections using the standard tissue-processing protocol. When injected into the dentate gyrus of adult mice, the type of retroviral vectors we used in this study are known to mainly transduce in newly generated neurons in the dentate gyrus (Fig. 2*B*). However, a modest number of non-neuronal cells are also infected to express transgenes. Under a fluorescence microscope, we were able to distinguish neurons from non-neuronal cells based on their morphology (Fig. 4*B*). The somata of labeled neurons were quickly identified and laser captured before being photo-bleached by excitation light. Figure 4*C* shows fluorescence images from sections before and after laser capture microdissection, which demonstrates the specific removal of the fluorescence signals in the somata of single neurons while maintaining surrounding tissue intact.

### Successful cDNA preparation and qPCR analysis

We pooled 150 and 300 laser-captured, single neuronal somata and extracted RNA. From these neuronal somata, we acquired 350 and 900 pg of RNA samples, respectively. These yields of RNA are sufficient for reverse transcription and cDNA amplification using the GeneChip WT Pico Kit, which requires a minimum of a 100 pg sample input. We used 270 pg of RNA as the sample input and acquired a yield of 77.8 μg of cRNA and 19.1 μg of single-strand cDNA (ss-cDNA). qPCR amplification of the cDNA for a housekeeping gene, β-actin, showed an amplicon at the expected molecular size on agarose gel electrophoresis (Fig. 4*D*). This result shows that the method for tissue processing and microdissection yields sufficient quality of RNA for downstream qPCR analysis. According to the manufacturer instruction, the cDNA yield we acquired is also sufficient for a potential application to perform microarray analysis (e.g., Whole-Transcriptome Array, Affymetrix). Using another sample of cDNA prepared in the same way, we confirmed that the successful laser capture collection of neurons by the amplification of the CaMKIIα (for excitatory neurons) and DCX (for newborn neurons; Fig. 4*D*).

### Evaluation of RNA quality

Our method uses conventional freezing, dehydration, laser capture, and RNA isolation procedures. There is no extra step to potentially reduce RNA quality, and therefore we expect that we can achieve the isolation of RNA in good quality, which is a requirement for the successful qPCR amplification described above. To confirm this expectation, we laser captured individual neurons in the granule cell layer one by one and pooled 5000 cells in each sample. Then we evaluated RNA integrity using Agilent RNA 6000 Pico Kit and 2100 Bioanalyzer (Schroeder et al., 2006). As described above, the yield of RNA collected from 100 to 300 cells was sufficient for successful downstream qPCR analysis but does not meet the minimum sample requirement for the RNA quality test. Therefore, we used larger samples by pooling 5000 cells for this analysis.

**Figure 5.**
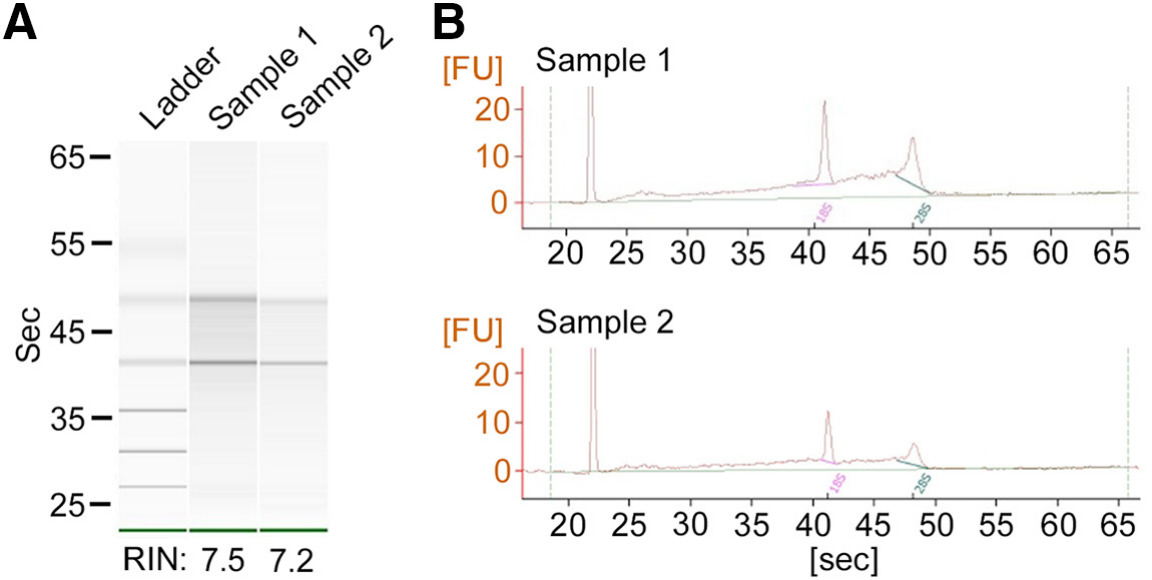
RNA quality assay. ***A***, Electrophoresis images of RNA samples. Clear bands of 18S and 28S ribosomal RNAs are visible. Ladder: 0.2, 0.5, 1.0, 2.0, 4.0, and 6.0 kb. ***B***, Electropherogram showing the distinct peaks of 18S and 28S ribosomal RNAs. FU, Fluorescence unit; Pink and thick green solid lines, baseline for 18S and 20S peaks, respectively; thin green solid line: overall baseline.

As shown in the electrophoresis images and electropherogram in Figure 5, two distinct bands of 18S and 28S ribosomal RNA were detected. RNA integrity numbers for two samples reached 7.5 and 7.2 (Schroeder et al., 2006). These values indicate a good quality of RNA, which is typically obtained by laser capture methods and is sufficient for downstream qPCR and transcriptomic applications (Butler et al., 2016; Ong et al., 2020). Thus, we validated that our hydration, laser capture, and RNA isolation method produce good-quality RNA.

### Specificity of microdissection

**Figure 6.**
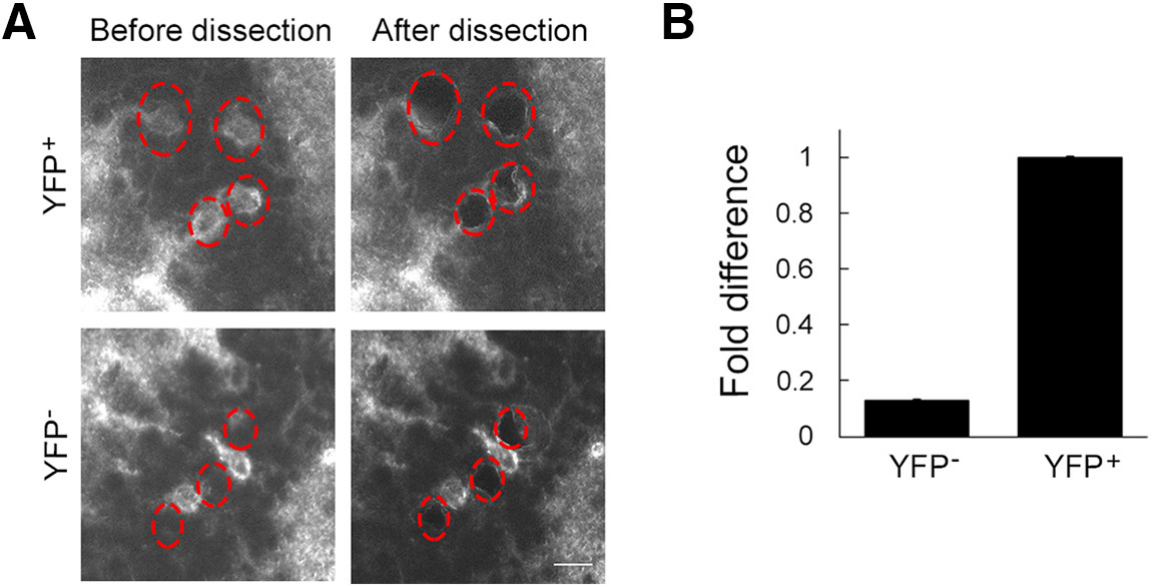
Evaluation of specific RNA collection from neurons targeted by laser capture microdissection. ***A***, Images of brain sections sparsely containing YFP^+^ neurons in the dentate gyrus. The brain sections were prepared from Pomc-ChR2-YFP mice. YFP^+^ neurons (top) and their neighboring YFP^−^ cells (bottom) were visually selected for microdissection (red dotted ovals). Scale bar, 20 μm. ***B***, Relative amount of YFP RNA in YFP^+^ neurons and their neighboring YFP^−^ cells, quantified by qPCR analysis.

The specificity for microdissection of single neuronal somata can be an issue since a targeted cell and its neighboring cells are often in close proximity. In addition, the typical thickness of brain sections (10–15 μm) used for laser capture microdissection allows multiple neuronal soma overlap in *z*-direction. Therefore, a certain degree of contamination during the isolation is unavoidable. To evaluate specificity of microdissection, we examined YFP gene expression in visually identified YFP^+^ neurons and YFP^−^ cells. For this experiment, we used POMC-ChR2-YFP mice that express ChR2-YFP in ∼10% of granule cells in the dentate gyrus. YFP^+^ granule cells and neighboring YFP^−^ cells were individually captured (Fig. 6*A*). The identity of YFP^−^ cells was not confirmed, but most of them are likely granule cells because we selected them from the granule cell layer. Approximately 300 cells were pooled for each sample of YFP^+^ and YFP^−^ neurons. Collected RNA was reverse transcribed and amplified into cDNA with GeneChip WT Pico Kit. The amplification yield of 107 μg (YFP^+^) and 102 μg (YFP^−^) of cRNA and 16.9 μg (YFP^+^) and 16.6 μg (YFP^−^) of ss-cDNA. From the amplified cDNA, the amount of YFP mRNA was quantified by qPCR. The result indicates there was as little as 10% of YFP mRNA in YFP^−^ cells compared with the amount in neighboring YFP^+^ granule cells (Fig. 6*B*), indicating high specificity in microdissection of single neuronal somata.

### Application of the technique to validate retrovirus-mediated single-cell gene knockout

Virus-mediated expression of Cre recombinase has been used to achieve sparse, single-cell gene knockout (Tashiro et al., 2006a,b). Validation of such sparse gene knockout is not straightforward because unaffected wild-type cells are intermixed. We applied our method of laser capture microdissection with fluorescent proteins fused to transmembrane proteins to validate that such virus-mediated single-cell gene knockout affects the expression of a target gene.

**Figure 7.**
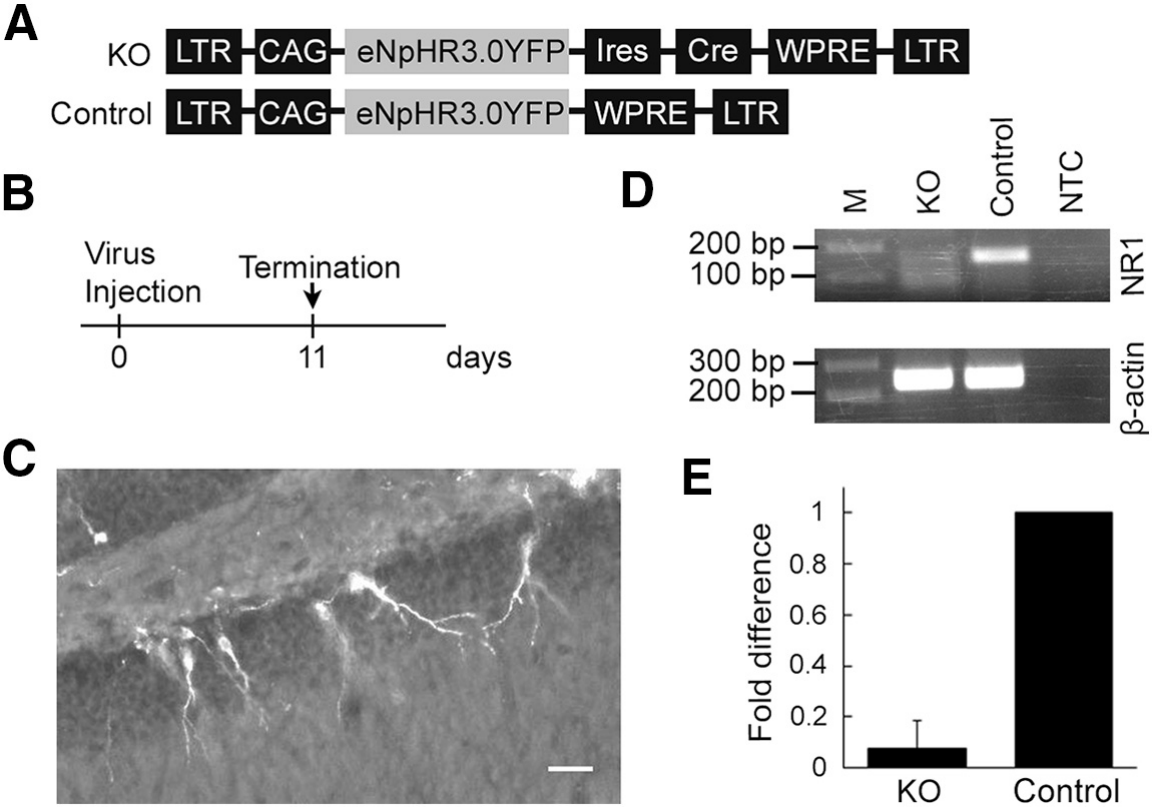
Validation of retrovirus-mediated, single-cell gene knockout in adult-born neurons in the dentate gyrus. ***A***, Schematic structures of the viral vector genomes. ***B***, Experimental time line. ***C***, Images of the dentate gyrus of floxed NR1 mice, which was injected with a bicistronic retroviral vector expressing eNpHR3.0-YFP and Cre. Scale bar, 50 μm. ***D***, qPCR amplification end products in gel electrophoresis. M, Molecular marker; KO, NR1 knock-out neurons with Cre expression; Control, control neurons without Cre expression; NTC, nontemplate control. ***E***, qPCR quantification of NR1 RNA in Cre-expressing (KO) and control adult-born neurons. Note the ∼92% reduction in Cre-expressing neurons.

We used transgenic mice with the floxed NR1 gene, in which the expression of Cre recombinase results in the deletion of NR1 gene (also called GluN1) encoding a subunit of NMDA receptor (Tashiro et al., 2006a). We injected these mice with a bicistronic retroviral vector expressing YFP-fused enhanced halorhodopsin and Cre (RV-CAG-eNpHR3.0-YFP-IRES-Cre) to knock out the NR1 gene. For control, transgenic mice were injected with a retroviral vector expressing YFP-fused enhanced halorhodopsin only. The vector constructs were described in Figure 7*A*. Brain sections were prepared 11 d after virus injection (Fig. 7*B*,*C*). For each sample, we captured >100 YFP^+^ neurons and pooled them for RNA extraction. We prepared four samples each for control and NR1 knockout. Extracted RNA was reverse transcribed and amplified into cDNA with a GeneChip WT Pico Kit. The amplification yielded 63, 101, 203, and 197 μg (control), and 62, 102, 178, and 210 μg (NR1 knockout) of cRNA; and 14.4, 15.2, 14.4, and 14.5 μg (control), and 14, 15.6, 14.6, and 14,5 μg (NR1 knockout) of ss-cDNA. qPCR analysis showed a 92.2 ± 11.0% reduction of the NR1 gene in the NR1 knock-out neurons compared with the control neurons (Fig. 7*D*,*E*), validating the successful removal of the NR1 gene in Cre-expressing granule cells.

## Discussion

In this study, we demonstrated that genetic labeling with fluorescent proteins fused to transmembrane proteins allows for the selective collection of genetically labeled fluorescent neurons using laser capture microdissection. Fluorescent proteins fused to transmembrane proteins preserve fluorescence signals in sections prepared from freshly frozen brains after the standard tissue-processing procedure for laser capture microdissection. Stable fluorescence signals allowed us to distinguish neurons from non-neuronal cells and laser capture hundreds of neurons easily. We successfully isolated RNA from the collected neuronal somata and determined that ∼100 cells are sufficient to obtain samples required for downstream applications such as qPCR and DNA microarray analysis.

Compared with conventional protocols of laser capture microdissection, observation and selection of fluorescently labeled cells are the only additional step. This additional step can be easily performed as far as a genetic method is available to label a cell type of interest with fluorescent proteins fused to transmembrane proteins. While this requirement may be a practical limitation for some applications, recent popularity in the use of optogenetic transmembrane proteins (Deisseroth et al., 2006; Pastrana, 2011; Pathak et al., 2013), which are often fused to fluorescent proteins, lowers a hurdle for this requirement. Plenty of transgenic and viral tools expressing channelrhodopsin or halorhodopsin are available commercially and/or from public depositories such as Addgene (http://www.addgene.org), which makes it relatively easy to express fluorescent proteins fused to transmembrane proteins in any neuronal (or other cell) types of interest.

We showed that fluorescent proteins fused to three different types of transmembrane proteins, ChR2, eNpHR, and TRPV2, can preserve fluorescence signals. ChR2 and TRPV2 are ion channels formed by multiple subunits with multiple transmembrane regions. eNpHR is an ion pump, but its structure is similar to that of ChR2. Thus, it is important to note that we tested relatively similar types of transmembrane proteins; therefore, it is not clear how much our result would be extended to other types of transmembrane proteins, for example, those with a single transmembrane domain.

We do not know why fluorescent proteins fused to those three transmembrane proteins can preserve fluorescence signals, but no regular or membrane-targeted protein does. However, it is known that freeze–thaw cycles damage the plasma membrane, and that soluble proteins, such as hemoglobin, are released from cells (Lovelock, 1953). Regular cytoplasmic fluorescent proteins are likely to diffuse out from cell bodies in this way. Transmembrane proteins are anchored tightly with and may stay with the plasma membrane, while the link with membrane-targeted fluorescent protein might not be strong enough to keep them associated with the membrane.

To validate the specificity of the RNA collection from selected cells, we used transgenic mice expressing ChR2-YFP and laser-captured YFP^+^ and YFP^−^ cells separately. qPCR analysis showed a 10-fold difference in the amount of YFP mRNA between YFP^+^ and YFP^−^ cells, demonstrating the high specificity of RNA collection using this method, although a small amount of contamination from neighboring cells may not be avoidable.

We applied this method to demonstrate the effect of gene removal by viral expression of Cre recombinase in floxed NR1 mice. A bicistronic viral vector expressing eNpHR-YFP and Cre was injected into the dentate gyrus of adult mice, and we laser captured YFP^+^ cells. The amount of NR1 mRNA in these YFP^+^ cells was reduced by up to 92% compared with wild-type neurons. Thus, this experiment provides an example of successfully applying the technique to an actual experiment. This method would be useful for similar experimental needs in validating single-cell genetic manipulations (Zong et al., 2005; Judkewitz et al., 2009; Luo et al., 2016; Kaneko, 2017; Nishiyama et al., 2017).

One useful application of this method would be to examine gene expression in different neuronal types. This sort of gene expression study has been successfully performed using genetic labeling with regular cytoplasmic fluorescent proteins and fluorescent-based sorting. However, genetic labeling often marks multiple neuronal types and/or other cell types (van Praag et al., 2002; Balthasar et al., 2004; Lagace et al., 2007; Harris et al., 2014), which makes it difficult to achieve RNA collection from a single neuronal type. For example, while the expression of parvalbumin is often used to genetically define a subtype of interneurons, it is well known that parvalbumin-expressing interneurons contain multiple neuronal types defined morphologically, including basket cells and axo-axonic cells (Hu et al., 2014; Nassar et al., 2015). In the conventional fluorescence-based sorting, these neuronal types cannot be separated. However, using our method, one should be able to separately laser capture these two morphologically distinct neuronal types and to analyze the gene expression of each neuronal type. One caveat for this application is a requirement of relatively sparse labeling to allow for visualization of the dendrites of individual neurons. For example, in Pomc-ChR2-YFP mice that we used in this study, ∼10% of granule cells express ChR2-YFP (Uemura et al., 2021), and we can clearly observe their dendrites (Fig. 2*C*). However, in another line of Pomc-ChR2-YFP mice that we used in a different study, nearly all granule cells express ChR2-YFP, the whole molecular layer of the dentate gyrus, which is occupied by granule cell dendrites, are filled with YFP fluorescence (Åmellem et al., 2021). In this case, we cannot visualize the dendritic morphology of individual neurons. Therefore, not all fluorescence-labeling methods work for this approach, and one may need to select a method for relatively sparse labeling (Zong et al., 2005; Badea et al., 2009; Judkewitz et al., 2009; Luo et al., 2016; Kaneko, 2017; Nishiyama et al., 2017).

Some morphological classification of neuronal types is largely subjective, which may make it difficult to reach agreement on classification among individual researchers. Or even if they are visually clearly distinguishable, there is no common ground for judging whether they should be classified as separate neuronal types. We believe that our method could provide a solution for this issue by giving an independent rationale for such “subjective” classification criteria. For this purpose, one needs to apply a subjective morphological classification and to perform gene expression analysis of those neuronal types using our method. If the two neuronal types have a clear difference in gene expression, one can conclude that they are two different neuronal types. If they do not have a difference in gene expression, one can conclude that they may not be different neuronal types. Furthermore, if some genes are expressed only in one of two neuronal types, one can use them as a marker for the neuronal type, which would make it easier to further confirm and use the classification in future studies. When it is difficult to make clear morphological criteria, one can test multiple subjective criteria. When the criteria separate two neuronal types better, the difference in gene expression would be clearer. Based on this idea, one determines the best version of morphological classification in terms of a difference in gene expression. Our method should be helpful to provide such tentative classification of neuronal subtypes based on morphology with an independent basis/rationale in terms of gene expression.

Related to the importance of morphological visualization, fluorescent proteins fused to transmembrane have an additional advantage. Compared with cytosolic fluorescent proteins, fluorescent proteins fused to transmembrane fluorescence proteins are distributed along cellular membranes and visualize small processes better than cytosolic fluorescent proteins (Moriyoshi et al., 1996; Fig. 2*B*). Such clearer visualization of dendritic morphology facilitates the identification of neuronal types.

In addition to gene expression profiling, laser capture collection of a selective neuronal population may be applied to different types of genomic analyses. Such applications include chromatin immunoprecipitation sequencing (seq) to identify binding sites for DNA-binding proteins (Park, 2009), the assay for transposase-accessible chromatin-seq to map chromatin accessibility (Buenrostro et al., 2013), and ChIA-PET, Hi-C, and HiChip to examine tridimensional chromatin conformation (Fullwood and Ruan, 2009; van Berkum et al., 2010; Mumbach et al., 2016).

Our study demonstrated the stability of fluorescence signals of fluorescent proteins fused to transmembrane proteins, which allows for selectively collecting a cell type of interest with laser capture microdissection. We validated that the RNA sample isolated with this method is suitable for downstream gene expression analysis experiments such as qPCR. The isolation of single neurons is specific with minimum contamination from neighboring cells. This methodology provides a novel alternative approach for gene expression analysis targeting a specific neuronal/cell type, which is especially advantageous when the specificity of genetic labeling is not sufficient and additional selection based on morphology is required.

## Synthesis

Reviewing Editor: Mikko Airavaara, University of Helsinki Decisions are customarily a result of the Reviewing Editor and the peer reviewers coming together and discussing their recommendations until a consensus is reached. When revisions are invited, a fact-based synthesis statement explaining their decision and outlining what is needed to prepare a revision will be listed below. The following reviewer(s) agreed to reveal their identity: Jose López-Atalaya.

First, I would like to thank you for submitting your manuscript to us. I have to apologize for the long time it took us to evaluate your study. I would like to remind that our focus is to publish results that are well validate and the conclusions are in line the data, and supported by statistical analysis. There were two reviewers in the manuscript and I reviewed the manuscript. The reviewers found your study potentially interesting. The value of this manuscript resides in a methodological improvement that may help neuroscience community interested in investigating gene expression in specific cell populations. The paper is well written and describes the methodological approach in sufficient detail to understand the work, and the manuscript is adequately referenced. However, the reviewers also raised several limitations and concerns that need to be addressed.

As shown in the manuscript, the approach can also be useful to laser capture transduced cells vs adjacent non-transduced cells when the neurons are constituents of a very homogeneous neuronal population such as the dentate gyrus of the hippocampus. However, the use of this approach to distinguish between neuron types is challenging since despite the vast amount of anatomical studies reported over the years, morphological classification of neuron types remains largely subjective and even for neurons that are visually distinguishable, there is no common ground to consistently define morphological types, and the authors did not address this concern.

The manuscript describes how authors encountered well-known difficulty regarding the loss of signal from fluorescent proteins during some fixation procedures. The authors observe that YFP fused to channelrhodopsin-2 or enhanced halorhodopsin, transmembrane proteins are not lost during ethanol only fixation, as compared to cytoplasmic mCherry or membrane attached GFP (GFP-F). However, retaining of fluorescent proteins fused to transmembrane proteins have been described in the literature ( doi:10.1084/jem.20061852.). Therefore advantages of tagging with membrane protein are known, although such fusion fluorescent proteins maybe not have been explicitly compared for utility in laser capture microdissection (LCM) in literature.

The study is limited in that the authors did not comprehensively demonstrate advantages of proposed utilization of proposed fluorescent tag and fixation method, nor do they compare sufficiently different types of fusion proteins that could be utilized.

The authors claim that their method allows them to obtain high-quality RNA additionally allowing for the selection of neuronal populations based on morphological analysis. However, the authors don’t demonstrate the high quality of RNA. The RNA quality was not assessed. Therefore we can’t determine advantages over methods that rely on the modified fixation with PFA ( doi:10.1371/journal.pone.0001151. ).

While staining was used to detect neuronal from non-neuronal cells, this was not validated trough e.g., gene expression analysis of specific cell type markers. This should be demonstrated.

Some comparison to non-membrane bound fluorescent proteins was done, it was not systematic enough. The authors used different fluorescent proteins (transmembrane bound YFP vs mCherry and GFP), expressed from different vectors (actually vectors for mCherry and GFP-F are not described). Also the investigation was limited to only two similar transmembrane proteins - channelrhodopsin and halorhodpsin fused with YFP.

## Author Response

Manuscript Instructions

- The species studied is not mentioned in the abstract. Please make sure to update both the abstract in the article file and on the submission form.

We added the species (mice) in the abstract (Ln 14) and on the submission form.

- The sex of the species studied is not mentioned in the materials and methods section. Please mention “male", “female", “x males and x females", “of either sex”.

We added the sex of the species (either sex) in the materials and methods section (Ln90).

---------------------------------------------

Synthesis of Reviews:

Comments on the Visual Abstract for Author (Required):

Is needed when accepted.

Synthesis Statement for Author (Required):

First, I would like to thank you for submitting your manuscript to us. I have to apologize for the long time it took us to evaluate your study. I would like to remind that our focus is to publish results that are well validate and the conclusions are in line the data, and supported by statistical analysis. There were two reviewers in the manuscript and I reviewed the manuscript. The reviewers found your study potentially interesting. The value of this manuscript resides in a methodological improvement that may help neuroscience community interested in investigating gene expression in specific cell populations. The paper is well written and describes the methodological approach in sufficient detail to understand the work, and the manuscript is adequately referenced. However, the reviewers also raised several limitations and concerns that need to be addressed.

Thank you for your positive evaluation of our manuscript. As described below, we addressed the reviewers’ concerns.

As shown in the manuscript, the approach can also be useful to laser capture transduced cells vs adjacent non-transduced cells when the neurons are constituents of a very homogeneous neuronal population such as the dentate gyrus of the hippocampus. However, the use of this approach to distinguish between neuron types is challenging since despite the vast amount of anatomical studies reported over the years, morphological classification of neuron types remains largely subjective and even for neurons that are visually distinguishable, there is no common ground to consistently define morphological types, and the authors did 2 not address this concern.

1) We agree that there is no common ground to consistently define morphological types even for visually distinguishable neurons. But this is the very reason why our method is important in the field of neuroscience. Using our method, one can test whether two visually distinguishable neuronal types have differences in gene expression. If they do not have difference, one can conclude that they may not be different neuronal types. If the two neuronal types have clear difference in gene expression, one can conclude that they are two different neuronal types. Further if some genes are expressed only in one of two neuronal types, one can use them as a marker for the neuronal type, which would make it easier to further confirm and utilize the classification in future studies.

2) We also agree that morphological classification of some neuronal types is difficult to reach agreement between individual researchers because morphological classification is often subjective. We believe that our method can also help this issue by giving an independent rationale for such “subjective” classification criteria.

For this purpose, one need to apply multiple different versions of “subjective” morphological classification. When the subjective criteria separates two neuronal types better, difference in gene expression would be clearer. Based on this idea, we determine which version of “subjective” morphological classification is best in terms of difference in gene expression. For this kind of analysis, our method is critical. Thus, the issue that the reviewers raised is indeed one of the reasons why our method is important in the field of neuroscience.

We added a paragraph (Ln 431-436) in discussion section.

The manuscript describes how authors encountered well-known difficulty regarding the loss of signal from fluorescent proteins during some fixation procedures. The authors observe that YFP fused to channelrhodopsin-2 or enhanced halorhodopsin, transmembrane proteins are not lost during ethanol only fixation, as compared to cytoplasmic mCherry or membrane attached GFP (GFP-F). However, retaining of fluorescent proteins fused to transmembrane proteins have been described in the literature ( doi:10.1084/jem.20061852.). Therefore advantages of tagging with membrane protein are known, although such fusion fluorescent proteins maybe not have been explicitly compared for utility in laser capture microdissection (LCM) in literature.

The article that the reviewer mentioned is “Selective depletion of Foxp3+ regulatory T cells induces a scurfy-like disease. Lahl K, Loddenkemper C, Drouin C, Freyer J, Arnason J,

Eberl G, Hamann A, Wagner H, Huehn J, Sparwasser T. J Exp Med. 2007 Jan 22;204(1):57-

63. doi: 10.1084/jem.20061852.” This study used transmembrane protein (diphtheria toxin receptor) fused to GFP, but we did not understand why the reviewers brought up 3 this article. However, we study the topic further and found an older article that showed that fluorescence from transmembrane protein fused to GFP is preserved after membrane permealization with ethanol, which is required for staining with propidium iodide and used in the article the reviewers indicated (PMID: 9415410 DOI: 10.1002/(sici)1097-0320(19971201)29:4<286::aid-cyto4>3.0.co;2-8).

We greatly appreciate the reviewers that they helped us find this piece of literature. We thought that other researchers must have seen similar phenomena. Therefore, when we prepared the first version of the manuscript, we made effort to find such a literature that show preservation of fluorescence in transmembrane protein fused to fluorescent proteins. However, unfortunately we failed. Now we are happy to find it with the reviewers’ help. And as the reviewers acknowledged, the novelty of our study is in the application of this phenomenon in laser capture microdissection and gene expression analysis.

By being inspired by the previous literature, we performed whether a freezing-thaw cycle also causes the disappearance of signal from regular cytoplasmic fluorescent protein in cell culture, in addition to in brain sections, and that this is the case. We also found cryoprotectant (10%DMSO/90%FBS) prevented the loss of fluorescence. We added this result as new Figure 3.

We added description of this previous study in introduction (Ln68-71).

The study is limited in that the authors did not comprehensively demonstrate advantages of proposed utilization of proposed fluorescent tag and fixation method, nor do they compare sufficiently different types of fusion proteins that could be utilized.

Thank you for constructive criticisms on our work. We addressed these issues as we describe individually below.

The authors claim that their method allows them to obtain high-quality RNA additionally allowing for the selection of neuronal populations based on morphological analysis. However, the authors don’t demonstrate the high quality of RNA. The RNA quality was not assessed. Therefore we can’t determine advantages over methods that rely on the modified fixation with PFA ( doi:10.1371/journal.pone.0001151. ).

In the original manuscript, we did not perform RNA quality analysis to keep the number of cells low (up to 300 cells). The yield of RNA collected from 100-300 cells was sufficient for successful downstream qPCR analysis, but does not meet the minimum sample requirement for the RNA quality test, and RNA quality analysis is difficult in such preparation (Foley et al., doi: 10.1101/gr.234807.118; Singh et al., doi: 10.1038/s41598- 019-41235-9; Chatzi et al., doi: 10.7554/eLife.45920.001).4

To confirm successful isolation of high quality RNA, we collected a larger pool of 5000 cells [similarly to what Khodosevich et al. 2007 (the article the reviewers mentioned) did] and evaluated RNA integrity using Agilent RNA 600 Pico Kit and Bioanalyzer 2100. Thus, although more cells/sections/mice are required, our method allows us to perform RNA quality analysis.

As shown in the electrophoresis images and electropherogram (new Figure 5A, B), two distinct bands of 18S and 28S were detected. RNA integrity numbers (Schroeder et al., 2006) reached 7.5 and 7.2 for two samples, which indicates good quality of RNA that is typically obtained by laser capture methods and sufficient for downstream qPCR and transcriptomics applications (Butler et al., 2016; Ong et al., 2020). Thus, we validated that our hydration, laser capture and RNA isolation method produces high quality RNA.

By the way, we would like to make sure with the reviewers that we do not intend to claim better RNA quality than Khodosevich et al. (2007) (the article the reviewers mentioned) or any other studies. Our main point is that our method complements other existing method. As far as labelling methods with transmembrane-fused fluorescent protein are available, our method allows one to avoid additional fixation procedures which often causes a problem in RNA quality.

While staining was used to detect neuronal from non-neuronal cells, this was not validated trough e.g., gene expression analysis of specific cell type markers. This should be demonstrated.

We used CaMKII alpha and doublecortin (DCX) genes as neuronal markers and show high expression in laser microdissected neurons. We thought that it is best to collect fluorescently-labelled neurons and non-neuronal cells, and compare them. However, our retroviral labelling only generates relatively low numbers of non-neuronal cells and we were not able to collect sufficient numbers of fluorescently-labelled non-neuronal cells.

Therefore, we compared neuronal marker gene expression between fluorescently labelled neurons and total liver RNA sample. This is presented as new Figure 2C. We shows that the neuronal markers are enriched in fluorescently labelled neurons collected with our protocol while these markers are undetectable in the liver RNA sample. Some comparison to non-membrane bound fluorescent proteins was done, it was not systematic enough. The authors used different fluorescent proteins (transmembrane bound YFP vs mCherry and GFP), expressed from different vectors (actually vectors for mCherry and GFP-F are not described). Also the investigation was limited to only two similar transmembrane proteins - channelrhodopsin and halorhodpsin fused with YFP.5

Regarding the part of the comment on the lack of vector description, we used retroviral vectors for mCherry and GFP-F in Figure 2. We described this at Ln 175-178 and 199-200 in the original manuscript, which unfortunately the reviewers may have missed. We believe that our manuscript was not clear enough in this respect. We apologize it. To improve it, we added labels in Figure 2 (RV: retroviral vector, TG: transgenic mouse, AAV: adeno-associated viral vector). We believe that this would make it easier to find the information and a section describing viral vector preparation in Materials and Methods.

2) We believe that whether comparisons are systematic or not is not a problem by itself. But we understand that the key point of the reviewers’ comment is that the reviewers have a concern in our conclusion that the transmembrane protein fusion made fluorescence signal of YFP and mCherry is preserved. More specifically, some other factors (e.g. types of vectors) may have made fluorescence signal preserved. And more systematic comparison would be one possible way to address such a concern. We believe that this is the key point of the reviewers’ comment.

We agree that more systematic comparison is more sophisticated and organized and it is easier to understand. However, it is only one possible way to reach the conclusion. We agree that our way shown in Figure 2 is less systematic and sophisticated, but we are sure that these results (with new results of TRPV2) in Figure 2 are sufficient to reach our conclusion that transmembrane protein fusion is a key for fluorescence preservation. We would like to explain this below.

Without fixation, fluorescent signal of regular fluorescent proteins is lost. This is a universally known phenomenon (as the reviewers acknowledged earlier part of this review “how authors encountered well-known difficulty regarding the loss of signal from fluorescent proteins during some fixation procedures”). There is not a dispute to the fluorescence signal of regular YFP, GFP and mCherry is lost if one does not fix the preparation. [Indeed, this is why the studies (including ours) showing specific methods overcoming the issue are seen valuable.]

Considering this, we do not believe that the lack of comparison e.g. between epHR3.0-

YFP vs YFP compromises the validity of our result. Important is that we found a new method to preserve fluorescence in freshly frozen sections; for example using the retroviral vector expressing eNpHR3.0-YFP. However, we agree that this finding with the retroviral vector expressing eNpHR3.0-YFP by itself does not lead us to our conclusion.

The requirement for fluorescence preservation may be transmembrane fusion (our conclusion) but alternatively can be the retroviral vector we used (alternative 1) or fusion to eNpHR3.0 (but not to any other transmembrane protein) (alternative 2). However, we showed that fluorescence is not preserved with the use of retroviral vector expressing mCherry (Figure 2A), which exclude alternative 1. We showed that fluorescence is preserved for ChR2-YFP (Figure 2B and D), which exclude alternative 2. One may want to consider more complex possibilities, for example a combination of a specific vector and 6 fusion to a specific protein is a requirement. But this possibility is also rejected by our new results that fluorescence signal is preserved when we used adeno-associated viral vector expressing mCherry-TRPV (different vector and different transmembrane protein).

Thus, we agree that more systematic comparison is more elegant and makes it easier to understand the conclusion. But its lack does not compromise the validity of our conclusion. We hope that the reviewers agree with that our current result is sufficient for our claim.

In addition, we would like to emphasize that in terms of vectors used, our results are systematic as we used the same retroviral vectors with different transgenes for Figure 2A, B, C and E. And now we included an additional result of ChR2(SSFO)-YFP in the same retroviral vector in Figure 2C.

3) To examine fluorescence preservation with the use of a different transmembrane protein, we used a transient receptor potential cation channel TRPV2 fused to mCherry.

We found that the fluorescence signal is preserved in Freshly frozen sections, showing that a different type of transmembrane protein also works for fluorescence preservation.

We added the result in Figure 2D
